# Noninvasive Imaging of Cancer Immunotherapy

**DOI:** 10.7150/ntno.50860

**Published:** 2021-01-01

**Authors:** Omar Abousaway, Taha Rakhshandehroo, Annick D. Van den Abbeele, Moritz F. Kircher, Mohammad Rashidian

**Affiliations:** 1 Department of Imaging, Dana-Farber Cancer Institute and Harvard Medical School, Boston, MA, 02215, USA.; 2 Department of Radiology, Brigham and Women's Hospital and Harvard Medical School, Boston, MA, 02215, USA.

**Keywords:** Noninvasive Imaging, Cancer Immunotherapy

## Abstract

Immunotherapy has revolutionized the treatment of several malignancies. Notwithstanding the encouraging results, many patients do not respond to treatments. Evaluation of the efficacy of treatments is challenging and robust methods to predict the response to treatment are not yet available. The outcome of immunotherapy results from changes that treatment evokes in the tumor immune landscape. Therefore, a better understanding of the dynamics of immune cells that infiltrate into the tumor microenvironment may fundamentally help in addressing this challenge and provide tools to assess or even predict the response. Noninvasive imaging approaches, such as PET and SPECT that provide whole-body images are currently seen as the most promising tools that can shed light on the events happening in tumors in response to treatment. Such tools can provide critical information that can be used to make informed clinical decisions. Here, we review recent developments in the field of noninvasive cancer imaging with a focus on immunotherapeutics and nuclear imaging technologies and will discuss how the field can move forward to address the challenges that remain unresolved.

## Introduction

Immunotherapy has revolutionized cancer treatment. Antibodies against immune checkpoint molecules such as programmed cell death protein 1 (PD-1) and its ligand PD-L1, and the cytotoxic lymphocyte antigen-4 (CTLA-4), temporarily blocks inhibitory signals of T cells, thereby strengthening activation of anti-tumor T cell responses 1,2. Checkpoint blockade treatments have resulted in durable responses in many cancers including melanoma and non-small cell lung cancer, among others 3,4. Adoptive cell therapy (ACT) has, likewise, showed great promise in the treatment of several malignancies, especially blood-born cancers 5. ACT uses expanded tumor infiltrating lymphocytes or engineered immune cells to induce an anti-tumor response 6,7. Initially, ACT involved *ex vivo* expansion of patients' tumor-infiltrating-lymphocytes (TILs) followed by reinfusion of expanded TILs 8. Later efforts have centered on engineering patient T cells to express a T cell receptor (TCR) or chimeric antigen receptor (CAR) that utilizes an antibody fragment, such as a single chain variable fragment (scFv), targeted to cancer specific markers. Cancer specific markers that have been targeted using CARs include CD19 and B cell maturation antigen (BCMA) on B cell malignancies, prostate specific membrane antigen (PSMA), and mesothelin 9,10. Recent preclinical and clinical efforts have successfully adapted ACT technology to other immune cells including Natural Killer cells (NK) and macrophages 11,12.

Notwithstanding the encouraging results, patient response to immunotherapy has been heterogeneous: while some patients show durable response, many patients only experience a partial or no response 13. Some patients also face serious immune-related adverse events (irAEs) such as dermatitis, colitis, endocrinopathies, hepatitis, pneumonitis, myocarditis and others which can be severe or even fatal 14,15. Therefore, stratifying patients with predictive markers prior to immunotherapy can potentially identify patients who will most likely respond to therapy, and avoid unnecessary toxicity in patients who are unlikely to respond. For example, it has been shown that the infiltration of T cells in the tumor microenvironment (TME) as well as the activation status of such T cells correlates with treatment outcome 16,17. Therefore, monitoring infiltration of T cells in patients holds predictive value. The gold-standard to predict response to treatment remains immunohistochemical staining of tumor biopsies. However, biopsies are invasive, prone to sampling errors, and single-tumor biopsy specimens may not reflect the immune response in the entire tumor burden due to extensive intra- and inter-tumor heterogeneity 18,19. Some lesions are not even accessible for biopsies 20, and, for obvious reasons, the number of lesions that can be biopsied in a patient in one session in a safe and reasonable manner is also limited. The fact that not every metastasis can be biopsied and combined with the knowledge that every metastases may harbor information crucial to designing the optimal treatment regimen, this illustrates the great need for more advanced imaging methods that can provide this information in an entirely noninvasive fashion.

Noninvasive functional whole-body imaging approaches, such as positron emission tomography (PET) and single-photon emission computed tomography (SPECT), combined as hybrid systems with computed tomography (CT) or magnetic resonance imaging (MRI) as PET/CT or PET/MRI are suitable for addressing this pressing need. PET relies on the detection of positrons emitted by radioisotopes, while SPECT relies on the detection of gamma particles. The most commonly used radiotracer is ^18^F-fluoro-2-deoxyglucose (^18^F-FDG), which is taken up by rapidly proliferating glucose-avid cancer cells and is used to detect primary and metastatic cancer lesions 21. However, ^18^F-FDG uptake is not tumor cell-specific as activated immune cells can also uptake ^18^F-FDG 22. Furthermore, tumors can be heterogeneous, and have distinct profile of infiltrating immune cells and cytokines. Therefore, to evaluate the response to immunotherapy, imaging strategies are needed to specifically image different subsets of immune cells and secreted factors. Numerous methods have been developed to address this issue. One approach is a method referred to as “immunoPET”, in which an antibody that targets a cell-surface marker specific for a cell type is radiolabeled with a PET isotope and used to track the dynamics of the targeted cells 23-25. The two common approaches used to image engineered T cells are *ex vivo* labeling of the cells with radioisotopes or the use of reporter genes that bind to targeted radiolabeled tracers 26,27. PET imaging through the use of radiolabeled cytokines and cytokine targeted antibodies such as IFN-γ in the TME has also shown promise as a tool to assess progress of immunotherapy in preclinical models 28. As the repertoire of immunotherapeutics expands rapidly, there is a great need to develop the tools to monitor, assess and even predict the response to these treatments. In this article, we will review the recent developments towards noninvasive whole-body imaging approaches to characterize tumors and assess the response to cancer immunotherapy.

## Small molecules for imaging immune responses

Radiolabeled small molecules comprise the largest group of PET tracers and are the most widely used for clinical PET scans 29. These tracers are based on drugs or metabolites with specificity for a desired cell type or metabolic process. Examples of such tracers include radiolabeled sugars, nucleosides, hormones, and small molecule drugs. These tracers are unique in their capacity to be transported through cell membranes to access intracellular protein targets, and thus enable targeting of a larger number of proteins. By virtue of their small size, small molecule tracers show faster clearance from circulation and higher tissue penetration relative to antibody-based tracers. Consequently, images can be acquired within minutes to hours after tracer administration.

Despite the favorable characteristics of small molecule tracers, there is difficulty in using them to assess the immune response to cancer. Because of the rapid metabolism and excretion of such tracers, there is typically high off-target uptake in the kidneys and bladder. Additionally, the most commonly used metabolism-based tracers cannot distinguish between activated immune cells and cancer cells. One such tracer, ^18^F-FDG, is an analog of glucose commonly used for cancer detection and diagnosis 30,31. ^18^F-FDG is also commonly used in patients receiving immunotherapy because of its ability to assess temporal changes in the entire tumor burden and its potential ability to diagnose immune related adverse events (irAEs) before they become symptomatic 32. ^18^F-FDG is taken up by metabolically active cells expressing the GLUT1 and/or GLUT3 transporters, which included both activated immune cells and cancer cells, as well as other glucose-avid cells such as the brain and heart 33-35. As such, ^18^F-FDG PET is unable to distinguish activated immune cells from cancer cells. Another example of a commonly used metabolism-based tracer for cancer diagnosis and detection is ^18^F-fluorothymidine (FLT) 36,37. ^18^F-FLT is an analog of thymidine and is phosphorylated by the enzyme Thymidine Kinase 1 (TK1). TK1 plays a key role in DNA replication and is highly expressed during the S phase of the cell cycle. Uptake of ^18^F-FLT reflects TK1 activity and cellular proliferation 36. Because ^18^F-FDG and ^18^F-FLT do not show selective uptake in immune cells, they are not suitable tracers for assessing the immune cells involved in the response to therapy with high specificity.

**Figure 1:**
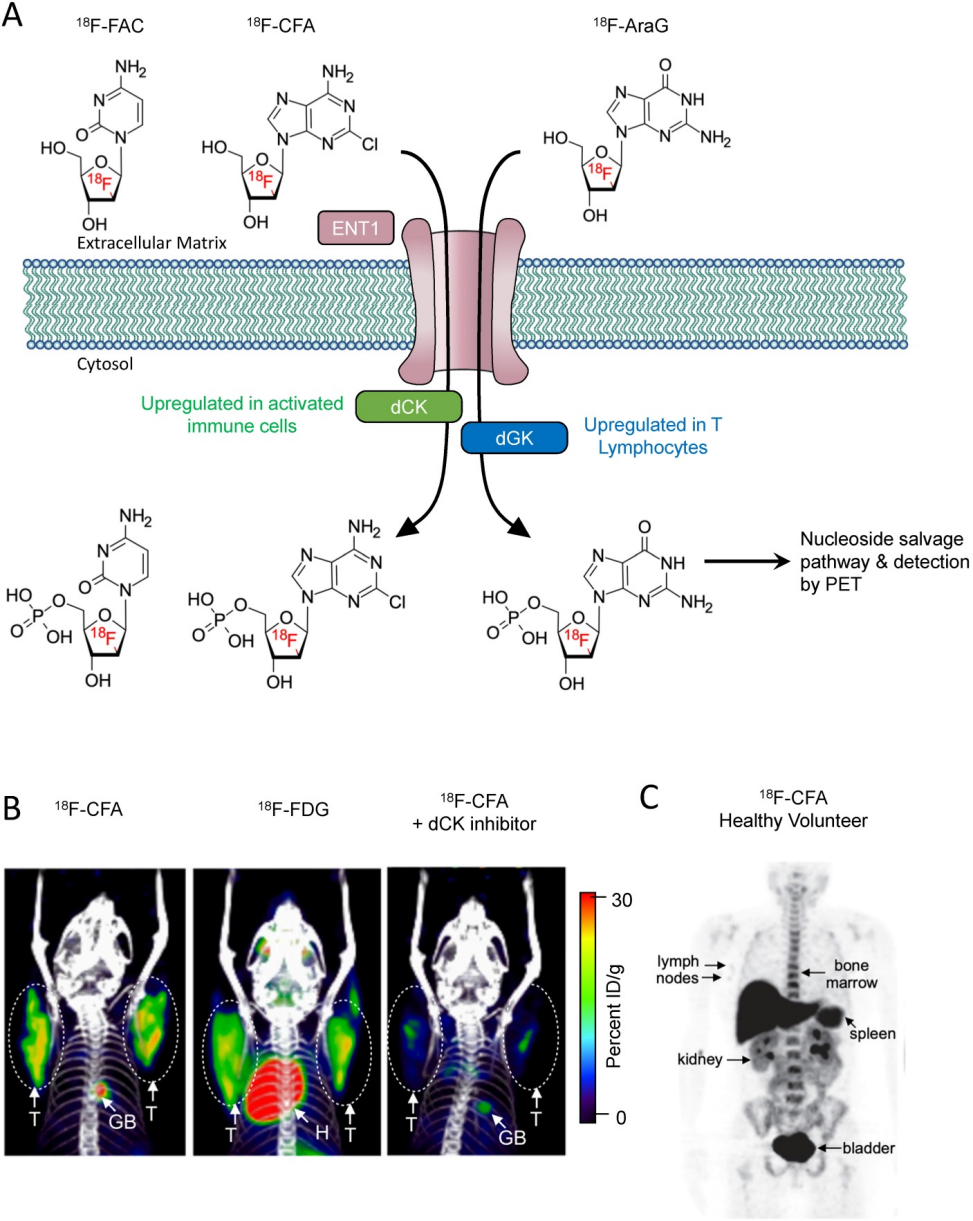
** Nucleoside analog-based PET imaging**. **(A)** PET tracers ^18^F-AraG and ^18^F-CFA are transported intracellularly by equilibrative nucleoside transporter 1 (ENT1). The tracers are then phosphorylated by their targets deoxyguanosine kinase and deoxycytosine kinase, trapping them intracellularly within cells. The tracers are then further metabolized along the nucleoside salvage pathway and incorporated into newly synthesized DNA.** (B)** PET/CT images of mice bearing CEM tumors, a human lymphoblastic leukemia, (circled) transduced to express Cytidine Deaminase (CDA). ^18^F-CFA was used in the left and right images, while ^18^F-FDG was used for the center image. A small molecule inhibitor of dCK, DI-82, was used in the right image to confirm the specificity of ^18^F-CFA for dCK. Adapted from 42. **(C)** ^18^F-CFA PET of a healthy human volunteer. Organs with high uptake are indicated with arrows. Adapted from 42.

Recently developed metabolism-based tracers take advantage of the metabolic pathways upregulated in immune cells to provide an immune cell-specific PET signal. While most tissues rely on the *de novo* DNA synthesis pathway, rapidly proliferating immune cells utilize the nucleoside salvage pathway for DNA synthesis 38. Deoxycytidine kinase (dCK) is the rate-limiting enzyme in the nucleoside salvage pathway, and is highly expressed in lymphocytes 39. Targeting dCK using radiolabeled small molecules is a strategy that has been used to selectively image immune cells (**Figure 1A**). ^18^F-FAC {1-(2'-deoxy-2'-[^18^F]fluoroarabinofuranosyl) cytosine} is a nucleoside analog that was identified by screening for molecules that show high retention in proliferating CD8+ T cells 40. During *in vivo* PET scans, ^18^F-FAC showed high signal-to-background in lymphoid organs as well as nonspecific uptake in the bone marrow and gastrointestinal tract. *Ex vivo* analysis revealed ^18^F-FAC labels T cells, B cells, and CD11b^+^ myeloid cells. In an oncoretrovirus tumor model, imaging with ^18^F-FAC showed high uptake in the tumor, tumor draining lymph nodes, and lymphoid organs 40. Because, ^18^F-FAC is rapidly catabolized by cytidine deaminase, it is likely not a suitable imaging tool for ultimate clinical translation 41. ^18^F-CFA (2-chloro-2'-deoxy-2'-[18F]fluoro-9-b-D-arabinofuranosyl-adenine) is a PET tracer designed to address this issue 42. ^18^F-CFA is also a substrate of dCK but is not catabolized by cytidine deaminase (**Figure 1B**) 42. In healthy patients, ^18^F-CFA shows high uptake in the lymphoid organs, as well as the liver and bone marrow (**Figure 1C**) 42. In a clinical study, ^18^F-CFA was used to assess the immune response to immunotherapy in glioblastoma multiforme (GBM) patients. After treatment with pembrolizumab and a dendritic cell vaccine, GBM patients showed increased uptake in the tumors as well as the secondary lymphoid organs. Importantly, uptake in the tumor correlated with the concentration of TILs 43. In an ongoing clinical trial, ^18^F-CFA is being used to assess the immune response to anti-PD-1 therapy in melanoma patients (NCT03409419). 9-(β-D-Arabinofuranosyl)guanine (AraG) is a guanosine analog with selective toxicity to T lymphocytes. Nelarabine is a prodrug of AraG that is used to treat T-cell acute lymphoblastic leukemia and T-cell lymphoblastic lymphoma 44. ^18^F-F-AraG was developed to selectively image T cells *in vivo* 45. Importantly, ^18^F-F-AraG does not result in toxicity to T cells *in vitro* 46. In a murine model of colorectal cancer ^18^F-F-AraG PET was able to successfully predict the response to anti-PD-1 therapy 48 hours after administration of anti-PD-1 therapy, responding mice showed a significantly higher PET signal in their tumors relative to non-responding mice 46. In a model of acute graft-versus-host disease, ^18^F-F-AraG showed high uptake in the secondary lymphoid organs prior to the manifestations of clinical symptoms. Uptake was also observed in the heart, spleen, and liver 47. In one of several ongoing clinical trials, ^18^F-F-AraG PET is being used to assess the response to checkpoint blockade therapy for patients with solid tumors (NCT03802123). In another clinical trial, ^18^F-F-AraG PET will be used to detect T cell activation in non-small cell lung cancer patients undergoing PD-1/PD-L1-directed therapy (NCT04186988).

The kynurenine pathway of tryptophan metabolism contributes to an immunosuppressive TME and has been a target for imaging. Tryptophan metabolites induce T cell apoptosis and upregulate the differentiation of Tregs *in vitro* 48,49. Macrophages and cancer cells express the enzyme indoleamine 2,3-dioxygenase (IDO) which metabolizes tryptophan to kynurenine 50. Expression of IDO is associated with poor clinical outcomes and increased cancer metastasis 51. 1-(2-^18^F-fluoroethyl)-L-tryptophan (1-L-^18^F-FETrp) is a PET tracer developed to monitor IDO-mediated tryptophan metabolism *in vivo* 52. In a preclinical mammary cancer model, 1-L-^18^F-FETrp showed high uptake in the tumor, by virtue of binding to IDO 52. This suggests that 1-L-^18^F-FETrp is a useful tool for assessing tryptophan metabolism *in vivo*, and may be helpful to assess the tumor immune landscape.

Small molecules that bind to markers of T cell activation can also be radiolabeled and used to assess the immune response to cancer. A ^68^Ga-radiolabeled granzyme B-binding peptide has been used to assess the response to checkpoint blockade 53. In a colorectal cancer model, mice were treated with both anti-CTLA-4 and anti-PD-1 antibodies and imaged 1 hour post-injection of the tracer. Mice that responded to treatment showed high uptake in the tumor, while non-responders showed low tumor uptake 53.

Small molecule tracers with specificity for immune cells have shown promise for predicting the response to immunotherapy. Small molecule tracers binding to immune cell markers such as CD8, CD4, CD11b, or secreted proteins such as IFN-γ, IL-2, or TNF-α could be highly useful for characterizing the tumor immune landscape with a reduced wait time post-injection.

## Radiolabeled antibodies and antibody fragments for imaging immune responses

**Figure 2:**
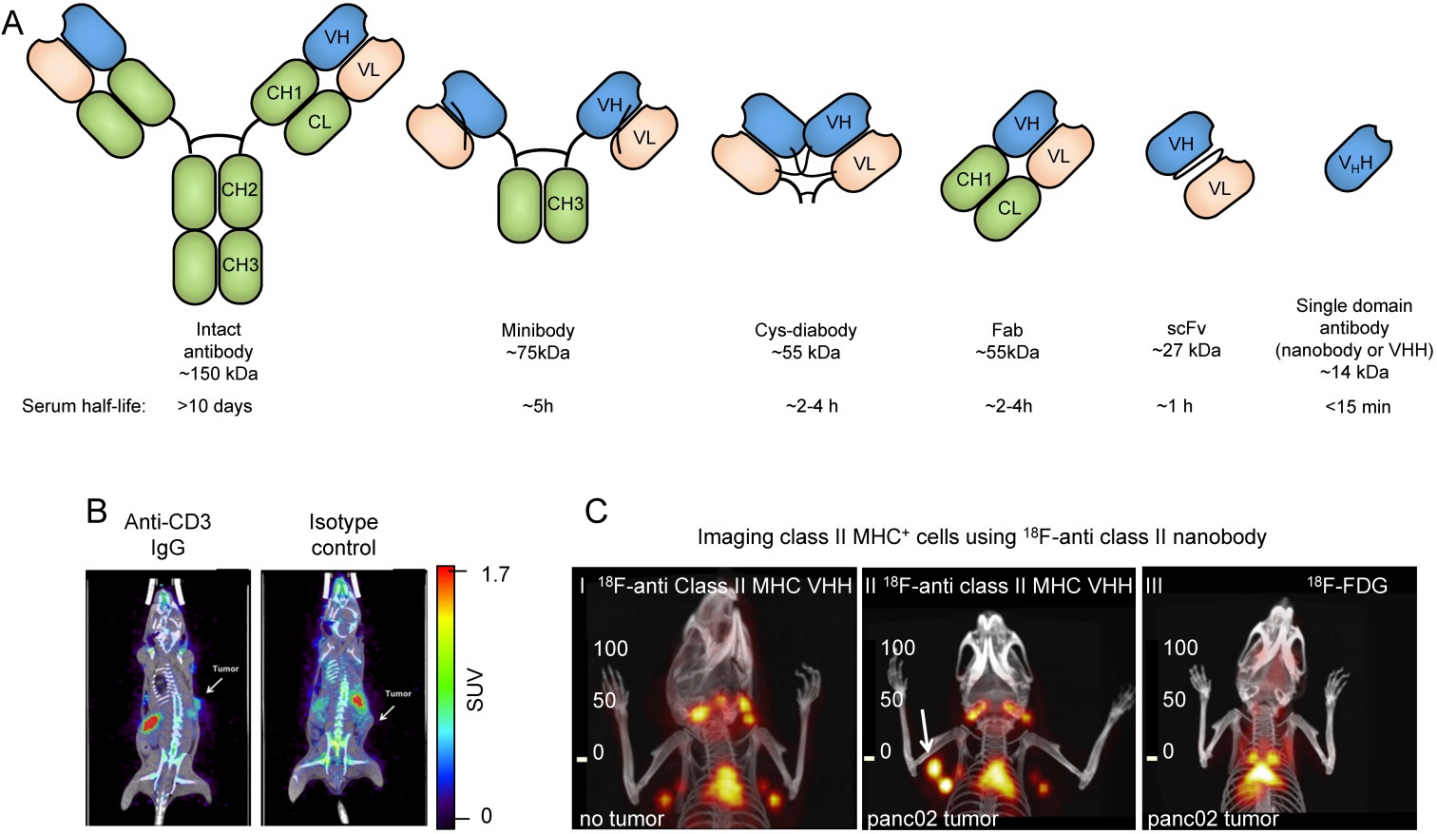
** Antibody-based tracers for *in vivo* PET imaging. (A)** Antibody fragments used for PET and SPECT imaging. **(B)** PET/CT images of mice bearing syngeneic BBN975 bladder tumors. The image on the left was acquired using an anti-CD3 IgG antibody-based tracer, while the image on the right was acquired using an isotype control IgG antibody. Images were acquired 72 h post injection of the tracer. Adapted from 73. **(C) C-I:** ^18^F-labeled anti-mouse class II MHC nanobody detects lymphoid organs (thymus and lymph nodes). **C-II:** The ^18^F-labeled anti-mouse class II MHC nanobody detects pancreatic panc02 tumor by virtue of detecting tumor-infiltrating class II MHC^+^ cells (white arrow shows the tumor). **C-III:** ^18^F-FDG fails to detect the same tumor. PET/CT images were acquired 2 h post-injection of the radiolabeled VHH or ^18^F-FDG. Data adapted from reference 64.

Due to the availability of antibodies against cell surface markers, and robust protein labeling techniques, imaging using radiolabeled antibodies has become a popular method for *in vivo* imaging of specific cell types. While intact antibodies have been used for imaging, their large size (~150 kDa) results in less than ideal imaging characteristics such as slow clearance from circulation (t_1/2_ of days to weeks), and relatively low tissue penetration. As a result, smaller antibody fragments such as minibodies, cys-diabodies, single chain Fvs (scFvs), and nanobodies have been developed and used for *in vivo* imaging (**Figure 2A**). The smaller size of these antibody fragments provides superior imaging characteristics, such as rapid clearance from circulation, higher tissue penetration, and higher signal-to-background ratios. Furthermore, these antibody fragments lack an Fc region, are not subject to recycling by the neonatal Fc receptor (FcRn), and, therefore, have a short half-life in circulation.

While antibodies and antibody fragments vary widely in their serum half-life, pretargeting radiolabeling methods have allowed for decoupling of the tracer distribution time and the radioisotope half-life necessary for imaging 54,55. In pretargeting, targeting antibodies or antibody fragments are labelled with a biorthogonal reactive group and injected into the subject. Then, at the desired time, the chelated radioisotope bearing a complementary reactive group is injected and allowed to react with the pretargeted vector. The subject is then ready for PET imaging. These methods are useful in cases where the targeting vector and radioisotope have incompatible half-lives (i.e. full length antibody serum t_1/2_ of days to weeks; ^18^F t_1/2_ of 109 minutes).

ImmunoPET has been used for *in vivo* imaging of cancer-specific markers, such as HER2 and EGFR 20,56. Detection of these markers has valuable applications in early detection of cancer, noninvasive tumor characterization, and guiding treatment decisions. Success in imaging cancer-specific markers in preclinical models has led to several clinical studies. In one such clinical trial, ^89^Zr-labeled trastuzumab was used to gauge the response of HER2^+^ breast cancer patients to an anti-HER2 therapy 57. Combined imaging before and after treatment had a 100% success rate in predicting patient response as assessed by time to treatment failure 57. While these results point toward promising applications of immuno-PET for tumor characterization and guiding targeted treatment, imaging of cancer-specific markers is usually unable to provide information assessing the tumor immune landscape or the immune response to immunotherapeutics.

Targeted imaging of immune cells can be used to assess the tumor immune landscape. Myeloid cells play key roles in shaping the immune status of the TME 58,59. Therefore, imaging their presence and activation status can help better assess the response to immunotherapy. Tumor-associated macrophages can significantly influence the TME immune landscape, for example by secreting different cytokines. M1-like macrophages secrete inflammatory cytokines such as CCL5, CXCL9 and CXCL10, which can recruit and activate T cells, whereas M2-like macrophages secrete cytokines that repel T cells. Thus, imaging the presence and phenotypic status of macrophages in the TME can provide valuable information for the assessment of immunotherapy response. A ^99m^Tc-labeled nanobody targeting the Macrophage Mannose Receptor (MMR, or CD206), which is highly expressed by the immunosuppressive M2-like macrophages, has been utilized for SPECT imaging in preclinical models of lung and mammary tumors. MMR^+^ macrophages were detected in hypoxic regions of the tumor with clarity in both cancer models, as early as 3 hours post-injection 60. Later PET imaging with a human/mouse MMR cross-reactive ^18^F-labeled nanobody in a mouse model of lung cancer validated MMR^+^-macrophage imaging 61. This anti-MMR nanobody has moved into clinical trial, where it will be used to detect tumor associated macrophages in patients (NCT04168528) 62. These studies indicate that imaging of MMR^+^ macrophages in the TME is feasible, and future studies will investigate whether it has predictive value for cancer immunotherapy.

CD11b is a myeloid cell marker that has served as a target for PET imaging of myeloid cells. An anti-CD11b ^99m^Tc-labeled intact antibody has been used for SPECT imaging of myeloid cells in a mouse colon cancer model. Animals showed high tumor uptake of the tracer indicating the presence of myeloid cells 6 hours post-injection 63. An anti-CD11b nanobody was also used to image tumor infiltration of myeloid cells in a melanoma model and could detect the tumor with clarity. Importantly, penetration of CD11b^+^ cells to the tumor core was found to be a negative predictor of tumor response to PD-1 blockade 64. Hence, CD11b-targeted PET could be a valuable tool for assessment of the TME in the clinic.

Professional antigen presenting cells, including macrophages, B cells, and dendritic cells, are pivotal to the development of an anti-tumor immune response. These cells are unique in their expression of MHC class II which is used to present tumor antigens to CD4^+^ T cells. A nanobody against mouse MHC class II was developed and used for *in vivo* detection of tumor infiltrating class II positive cells in both syngeneic and xenogeneic preclinical models. This ^18^F-radiolabaled nanobody could detect tumors with clarity, by virtue of detecting infiltrating class II positive cells as early as 2 hours post-injection 64,65. An anti-human class II nanobody was also developed and used to image class II positive cells in a humanized mouse model of Graft versus Host Disease (GvHD). Mice that developed GvHD showed high uptake of the anti-class II tracer in the liver, corresponding to an increase of MHC II^+^ immune cells 66. Furthermore, nanobody-based tracers specific for dendritic cells have been developed 67. While the importance of professional antigen presenting cells in the TME has been acknowledged, further studies are needed to determine whether *in vivo* imaging of this subset holds prognostic value.

Natural killer cells can mediate an anti-tumor response against cancer cells that down regulate MHC class I expression and are thus a valuable target for PET imaging. NKp30, a natural cytotoxicity receptor that is upregulated on activated natural killer cells has been used as a target for PET imaging 68. NSG mice, lacking autologous NK cells were injected with NK92MI cells, an immortalized NK cell line. Mice were later injected with a ^64^Cu-labelled anti-NKp30 and imaged 48 h later. Mice injected with NK92MI cells showed high uptake in the spleen, indicating an NK cell-specific signal 68. Other studies have assessed NK cell and NK CAR cell tumor infiltration by *ex vivo* labelling 69,70. Further studies are needed to explore to what extent immunoPET can be used to assess NK cell tumor infiltration.

Of particular interest is the imaging of T cell infiltration of tumors as a potential prognostic and predictive factor. Cytotoxic CD8^+^ T cells directly mediate much of the anti-tumor immune response induced by checkpoint blockade and, thus, are a potential target for tumor characterization and monitoring the progress of immunotherapeutics 71. Likewise, CD4^+^ T cells play an important role in shaping the TME, and monitoring their tumor penetration holds prognostic value 72. Several strategies have been developed to image T cells in both preclinical and clinical settings. This includes using antibody and antibody fragments targeting CD3, CD4 and CD8, specific markers of T cells, helper T cells and cytotoxic T cells, respectively. A ^89^Zr-radiolabeled anti-CD3 antibody was found to be able to detect tumor infiltrating lymphocytes in a syngeneic murine model, 72 hours post injection (**Figure 2B**) 73. *In vivo* imaging using a ^89^Zr-radiolabeled anti-CD4 cys-diabody resulted in high uptake in lymphoid organs, with favorable signal/background ratios, 20 h post-injection 74. Minibodies specific for mouse CD8 have been developed for PET imaging of CD8^+^ T cells. The ^89^Zr-radiolabeled minibodies resulted in high uptake in the lymphoid organs in mice, when imaged 4 hours post-injection 24. These studies demonstrate that *in vivo* imaging of T cells is feasible.

Other studies were able to correlate T cell infiltration into tumors with treatment outcomes. A ^89^Zr-labeled anti-CD3 antibody was used to assess the immune-mediated response to CTLA-4 blockade, 3 days post-injection of the tracer in a preclinical colorectal cancer model 75. After anti-CTLA-4 treatment, it was found that responders had higher T cell infiltration compared to nonresponders. The authors concluded that the anti-CD3 imaging could stratify responding tumors based on their T cell infiltration. A cys-diabody against CD8 was developed for the detection of tumor infiltrating CD8^+^ lymphocytes *in vivo* 76. In a murine model of colorectal cancer, mice were treated with an anti-PD1 antibody and imaged 48 hours later; images were acquired 22 hours post injection of the tracer. It was found that greater infiltration of CD8^+^ T cells into tumors was predictive of response to anti-PD1 therapy 76. A nanobody against CD8 was developed and used to detect CD8^+^ T cells *in vivo*. Addition of a 20 kDa polyethylene glycol (PEG) moiety to the nanobody significantly enhanced signal-to-background ratio and reduced uptake in the kidneys, potentially owing to its increased circulatory half-life and hydrophilicity 23. In a B16 melanoma model, mice were treated with anti-CTLA-4 antibody and vaccinated with irradiated B16 tumor cells engineered to secrete granulocyte-macrophage colony-stimulating factor (the vaccine is usually referred to as “GVAX”) 77. The response was monitored longitudinally by CD8 PET imaging once a week for 4 weeks. It was found that homogeneous distribution of CD8^+^ T cells in tumors was predictive of response to CTLA-4 blockade (**Figure 2C**) 23. In later work, the ^89^Zr-labeled PEGylated anti-CD8 nanobody was used to image T cell infiltration in a PD-1 responsive colorectal cancer model 78. Responding mice showed a significant increase in T cells and their penetration into the tumors, whereas nonresponding mice showed CD8^+^ T cells remaining mostly around the tumor periphery 78. Imaging of CD8^+^ cells has recently begun translation to the clinic. In a recent first-in-human study, a ^89^Zr-labeled anti-CD8 minibody was used to detect CD8^+^ T cell tumor infiltrates in patients with hepatocellular carcinoma, melanoma, and lung cancer (^89^Zr-labeled-IAB22M2C) 79. The tracer showed high uptake in tumors, as well as the liver, kidneys and spleen. Images were acquired 2-4h, 24 ± 4 h, 48 ± 4 h, and 92-148 h post injection. These studies show that infiltration of T cells into tumors is predictive of response to immunotherapy and can be assessed by immunoPET. This ^89^Zr-labeled-IAB22M2C tracer has now moved into a phase 2 A open label, multi-dose trial in patients with metastatic solid tumors prior to and after standard immunotherapy (NCT03802123).

T cells are likely the major contributors to the anti-tumor immune response of checkpoint blockade. However, not all T cells can kill tumor cells or contribute in reshaping the immune landscape of the TME to a more anti-tumor status. Activated T cells, however, are likely the major cells that contribute to the anti-tumor response of checkpoint blockade. Therefore, the ability to detect their infiltration into tumors holds great prognostic value. While detection of TILs through anti-CD3 or anti-CD8 antibodies has been used to predict the response to immunotherapy in murine models, detected T cells may be exhausted or anergic and not capable of actively mounting an anti-tumor response. An important attribute of activated T cells is their increased surface expression of costimulatory molecules such as ICOS, 4-1BB, and OX40 80-82. As such, one strategy to image activated immune cells is to use radiolabeled antibodies against these molecules. A recent study used a ^89^Zr-labeled intact antibody against ICOS to detect activated CD4^+^ and CD8^+^ T cells *in vivo* 83. In a lung cancer model, tracer uptake in the tumor and tumor draining lymph nodes predicted the tumor growth rate response to a stimulator of interferon genes (STING) agonist treatment, 2 days post-injection. In similar work, a radiolabeled anti-OX40 intact antibody was used to detect activated T cells *in vivo* 84. In mice bearing A20 B cell leukemia tumors, tracer uptake in the tumors and tumor draining lymph nodes 4, 16, and 24 hours after tracer administration, successfully predicted response to CpG vaccination. These results show that imaging of activated CD8^+^ T cells is a valuable tool for assessing the immune response to cancer immunotherapy.

The presence of tumor-specific T cells in the TME has been shown to be essential for mounting an effective anti-tumor immune response 85,86. Imaging antigen-specific T cells is thus of great importance. A recent study used a peptide-loaded ^64^Cu labeled MHC construct dimerized via an Fc fragment to image, *in vivo*, HPV and influenza-specific CD8^+^ T cells in the context of HPV-tumor-bearing and influenza-infected mice, respectively 87.

Taken together, a large body of preclinical data suggests that imaging immune cells by immuno-PET can be used to assess, prognosticate and even predict the response to cancer immunotherapy, some of which have already moved into clinical trials. In the future, we can anticipate the development of more preclinical approaches and translation of more of these efforts into clinical settings, where they will help guide clinical decision-making.

## Imaging cytokines

**Figure 3:**
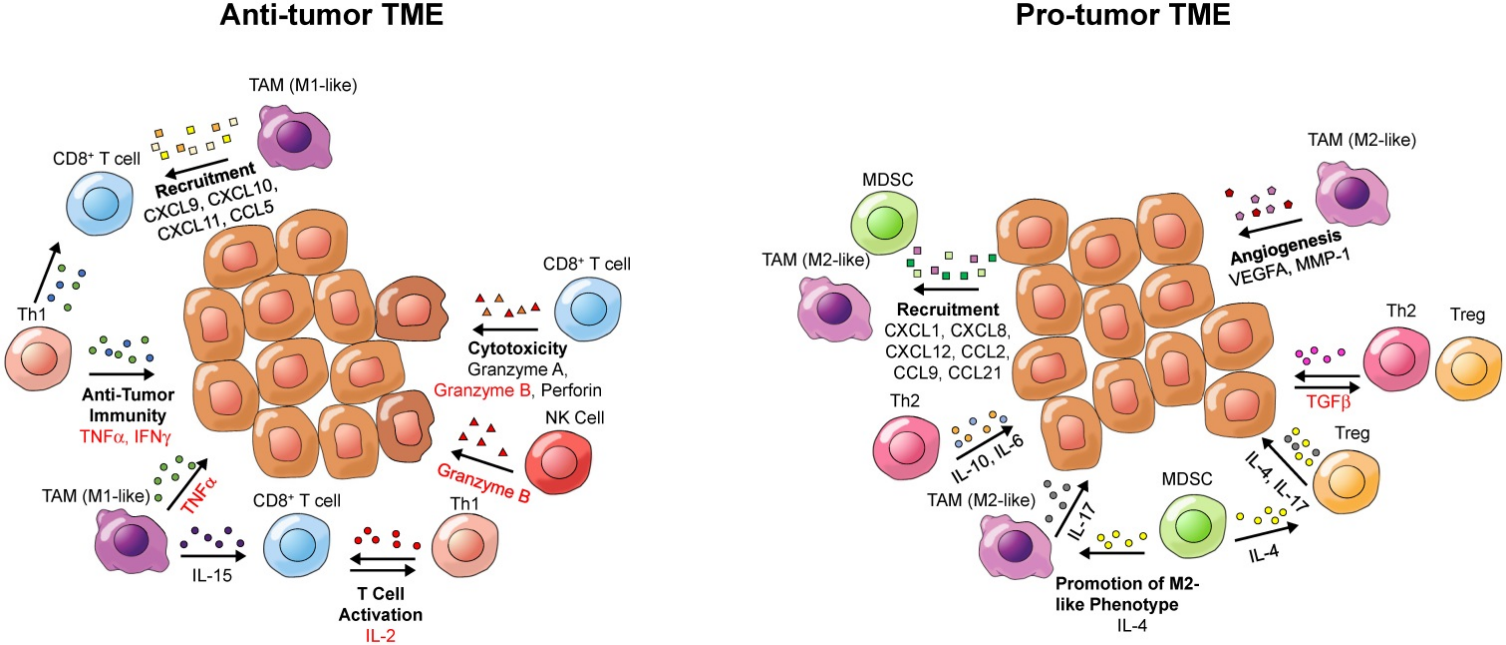
** Cytokines and potential imaging targets of the tumor microenvironment (TME)**. Depicted are cytokines present in either a pro-tumor or an anti-tumor TME, which could be targeted for tumor characterization by PET. Cytokine names that have been imaged in prior studies are written in red; cytokines that have not been previously imaged are written in black. Cytokine categories are written in bold. Arrows point from cells secreting cytokines toward target cells.

While defects in oncogenes and tumor suppressor genes underlie a genetic component of cancer, the microenvironment that tumors inhabit has a great influence on tumor growth and the immune-mediated response to cancer. Cytokines within the TME can contribute to an inflammatory or anti-inflammatory TME (**Figure 3**). Pro-inflammatory cytokines can contribute to an anti-tumor response by enhancing cytotoxicity, increasing tumor antigen presentation, recruiting T cells and inducing cancer apoptosis 88, while anti-inflammatory cytokines can induce an immunosuppressive tumor landscape 89. Therefore, detection of cytokines in the TME can provide information about the immune landscape of the tumor and allow for assessment or even prediction of the response to immunotherapy. Of note, circulating cytokines can also bind to the PET tracers, which may impair the detection of cytokines in the TME.

Several preclinical studies have been performed to noninvasively image cytokines (**Table 1A - Table 1E**). A ^89^Zr-labeled anti-IFN-γ antibody has been used to detect IFN-γ in the TME of a mammary cancer model 28. Mice were inoculated with HER2+ tumors and treated with a HER2-based DNA vaccine, encoding the extracellular and transmembrane regions of HER2 as well as GM-CSF, which upon secretion would help to recruit antigen-presenting cells and thus enhance tumor-specific T cell priming. Vaccinated mice showed higher uptake of the tracer in tumors, indicating increased antitumor immunity. The tracer uptake was negatively correlated with the tumor growth rate 28. In another study, the TNF-α inhibitor etanercept was radiolabeled with ^64^Cu and used for PET imaging of TNF-α in a model of acute and chronic inflammation 90. Mice were injected in the right ear with 12-O-tetradecanoyl-phorbol-13-acetate (TPA) to induce inflammation. After a single injection of TPA, tracer uptake significantly increased in the right ear, indicating increased inflammation.

PET imaging has also been used to assess the tumor penetration of cytokine-targeted antibodies in patients. In a clinical trial (NCT01472731), patients with recurrent high grade GBM were imaged with ^89^Zr-fresolimumab, a radiolabeled anti-TGF-β monoclonal antibody 91. The purpose of the study was to assess the tumor penetration and therapeutic efficacy of fresolimumab. ^89^Zr-fresolimumab showed high uptake in high-grade gliomas, though fresolimumab showed no clinical benefit. This study indicates that cytokine-targeted PET tracers are capable of penetrating patient tumors, and immunoPET may be able to detect cytokines in the TME of patients.

Another PET imaging strategy has involved the use of radiolabeled cytokines to detect activated T cells, or other immune cells, expressing their cognate receptors. Several studies have used Interleukin-2 (IL-2)-based tracers for detection of activated T cells expressing the IL-2 receptor 92,93. More recently, N-(4-[^18^F]fluorobenzoyl)-IL-2 (^18^F-FB-IL-2) has been used to detect activated T cells in the TME of a lung cancer model 92. Tumor bearing mice were treated with either tumor-specific vaccination or X-ray irradiation. Mice whose tumors were irradiated and those that received a vaccine showed greater uptake of ^18^F-FB-IL-2 in their tumors, indicating an increased presence of activated T cells. Notably, ^18^F-FB-IL-2 PET imaging has advanced to clinical trials (NCT02922283, NCT03304223, NCT04163094, NCT02478099). However, the first such clinical trial was terminated, as tracer uptake did not correlate with treatment outcomes (NCT02922283).

These data demonstrate that imaging of cytokines and their receptors can be useful for gauging the anti-tumor immune status of the TME. There is a need to better understand the dynamics of different cytokines, such as CCL5, CXCL9, CXCL10, TGF-β or IL-15, in the TME and their correlation with the treatment outcome. This, in turn, may help us to better assess or predict the response to immunotherapy, or guide clinical treatment decisions otherwise.

## Imaging checkpoint molecules

**Figure 4:**
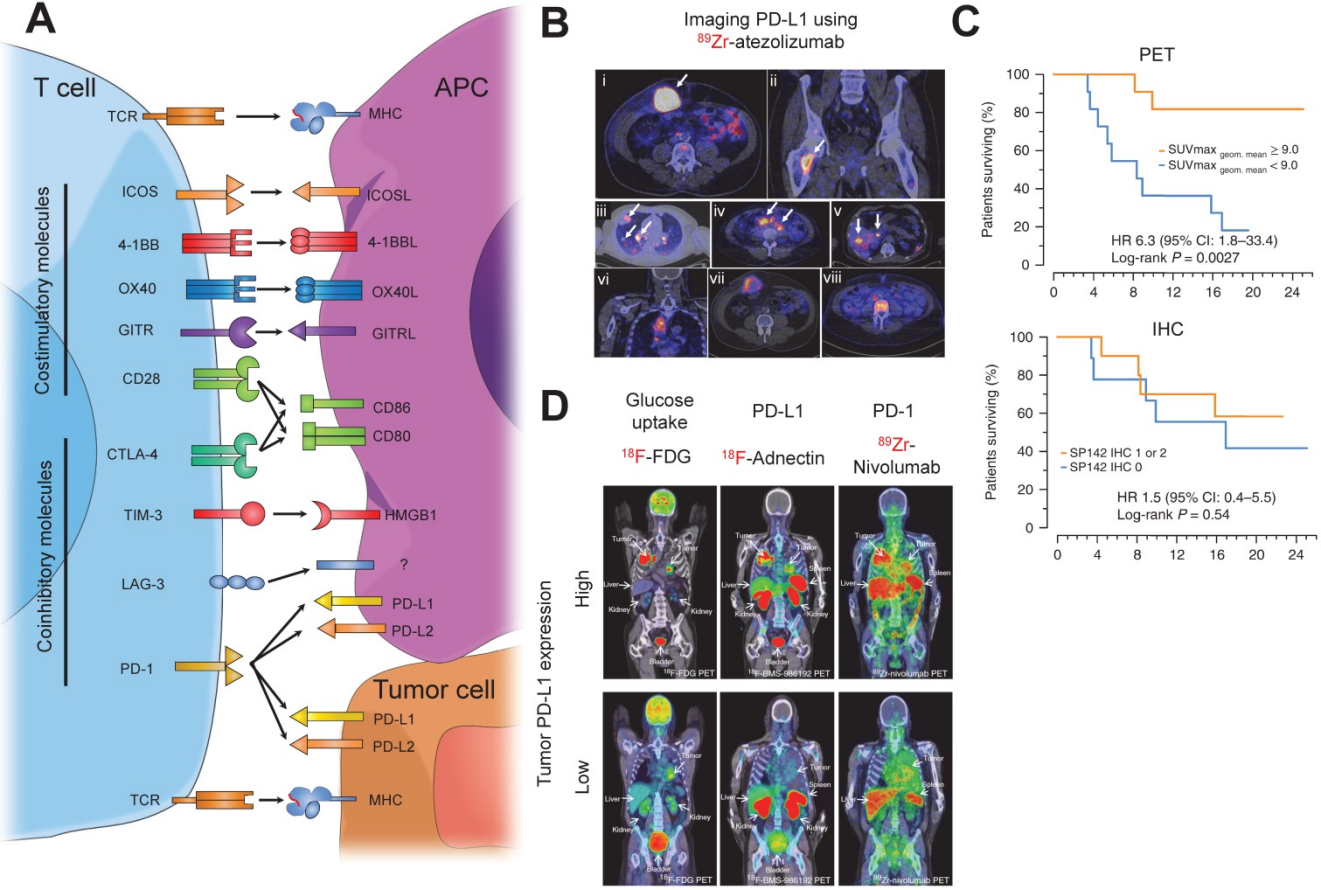
** PET imaging of checkpoint molecules. (A)** Schematic representing the major costimulatory and co-inhibitory molecules on a T-cell and their respective ligands that are expressed on either antigen presenting cells (APC) or tumor cells. **(B)** Representative images of PET images from bladder, non-small cell lung, and triple-negative breast cancer patients showing homogenous (i-v) and heterogenous (vi-viii) intratumoral labeling of PD-L1 using ^89^Zr-atezolizumab. Adapted from 112. **(C)** Kaplan-Meier curves taken from patients in (B) treated with PD-L1 blockade using atezolizumab after PET imaging. PET imaging of PD-L1 expression shows a much higher power to predict response to PD-L1 blockade compared to a clinically available IHC protocol. Adapted from 112. **(D)** Representative PET images of advanced non-small cell lung cancer patients infused with ^18^F-FDG, a marker for glucose uptake, ^18^F-BMS-986912, an anti-PD-L1 adnectin, and ^89^Zr-nivolumab, an anti-PD-1 IgG antibody, followed by nivolumab treatment. PET images indicate the anti-PD-L1 adnectin can target and label PD-L1 expression in the tumor while ^89^Zr-nivolumab imaging indicates T cell infiltration in tumors expressing PD-L1. Adapted from 120.

Immune checkpoint molecules such as PD-1 and CTLA-4 are inhibitory molecules that regulate activity of T cells (**Figure 4A**). Immune checkpoint blockade functions by temporarily blocking these inhibitory signals of T-cell activation. As a result, this enhances the tumor-reactive T cell's ability to mount an effective anti-tumor response. CTLA-4 blockade may result in depletion of regulatory T cells, which can further help the cytotoxic T cells to mount a stronger response against tumors. However, such depletion has only been observed in animal studies but not in patients treated with ipilimumab (IgG1) or tremelimumab (IgG2), the two FDA-approved anti-CTLA-4 antibodies 94.

Checkpoint blockade treatments have elicited favorable clinical responses in patients with melanoma, lung, head and neck, urothelial cancer, and Hodgkin lymphoma, among others 95. Being able to noninvasively assess through PET imaging, expression and location of these immune checkpoint molecules can provide a pathway to better clinical prognostic and predictive tools 96.

### PD-L1

PD-L1 can be expressed on cancer cells and immune cells and can directly interact with PD-1 molecules on T cells to inhibit T cell function. Studies have illustrated that expression of PD-L1 can be a predictive marker for response to PD-1/PD-L1 blockade in several cancers, including lung, breast, bladder, cervical, and gastric cancer 97. Therefore, determining expression of PD-L1 could serve as a clinically relevant predictive and prognostic marker for PD-1/PD-L1 blockade. The current clinical protocol to continue with anti-PD-L1 immunotherapy is determined by immunohistochemical (IHC) staining of biopsied tumor sections for PD-L1 expression 98,99. Although IHC is a well-established method in clinical practice, single biopsy samples, mis-sampling of the tumor, inability in obtaining adequate samples in patients with metastatic disease, variability in baseline expression, or the invasiveness of obtaining samples can all contribute to inconsistent and nonrepresentative results given extensive intra- and inter-tumor heterogeneity. However, whole-body noninvasive imaging platforms, such as PET that provided entire body and tumor burden visualization, quantification, and localization of expression throughout the body is more comprehensive, informative, and prognostic compared to IHC.

To determine the feasibility of imaging PD-L1, a preclinical PET imaging study targeting PD-L1 was conducted in C57BL/6N mice implanted with PD-L1 expressing B16 melanoma and imaged with ^64^Cu-NOTA-labeled anti-mouse PD-L1 IgG (clone 10F.9G2) 100. A 2-fold increase in tracer uptake to the tumor was observed compared to the PD-L1 knockout control tumor 24 hours post-injection. To further establish the relevance of PD-L1 imaging, a preclinical study was conducted assessing PD-L1 expression upon treatment. It has been shown that radiation therapy (RT) synergizes with PD-1 blockade in preclinical melanoma models 101 and head and neck squamous cell carcinoma (HNSCC) 102. To assess whether PD-L1 expression in the tumor increases in response to RT, a subsequent study treated syngeneic murine head and neck squamous cell carcinoma (HNSCC) and melanoma tumor bearing mice with RT. The animals were injected with ^89^Zr-DFO-anti-mouse PD-L1 IgG (clone 10F.9G2) and imaged 24 hours later. PET imaging as well as *ex vivo* analysis revealed a 2-fold increase in uptake of the targeted radiolabel in RT-treated tumors compared to untreated control, indicating PD-L1 induction can be visualized using PET imaging 103.

Preclinical studies have also utilized small proteins or single domain antibody fragments specific for PD-L1 due to their fast clearance from the blood and high tumor penetrance. For example, a nanobody against PD-L1 showed low background and high uptake in implanted melanoma tumors expressing PD-L1 after only 1 hour post-injection with peripheral organ uptake mainly limited to the kidney 104. Several other studies have also developed nanobodies targeting PD-L1 for the purpose of PET imaging with similar imaging and biodistributional profiles 105-107. Additionally, an anti-PD-L1 affibody 108, which is an engineered three-helix bundle based on the 58-residue (6.5 kDa) scaffold of the IgG-binding Z domain of staphylococcal protein A, 109 has been used as a PET tracer to image PD-L1 expression. This tracer showed a 3-fold increase in signal in tumors, compared to non-PD-L1 expressing controls only 2 hours post-injection. In the same study, a synthetic macrocyclic peptide specific for PD-L1 showed a 5-fold increase in signal in tumors relative to controls 109. *Ex vivo* biodistribution studies revealed that tracer uptake is limited to the tumor, kidneys, and liver. Although these small proteins show promise due to their fast clearance, many preclinical studies have utilized clinically-approved anti-PD-L1 antibodies as PET imaging agents due to their easy transition into the clinical space.

Atezolizumab, an FDA-approved anti-PD-1 IgG antibody, has shown promising results in the treatment of urothelial carcinoma and NSCLC 95. Preclinical studies using immune-compromised NSG mice with triple negative breast cancer (TNBC) tumor implants have shown ^111^In-labeled atezolizumab (SPECT imaging) 110 and ^64^Cu-atezolizumab (PET imaging) 111 uptake was 2-fold higher in PD-L1 high expressing cells compared to PD-L1 low expressing cells. *Ex vivo* biodistribution analysis revealed uptake of both tracers in the blood, spleen, kidney, liver, and lungs. This study facilitated the first-in-human clinical trials using ^89^Zr-labeled atezolizumab in patients with metastatic bladder cancer, non-small cell lung cancer (NSCLC), and TNBC 112. Biodistribution studies indicated tracer uptake in intestines, kidney, and liver, reflecting locations of antibody metabolism and elimination as well as the spleen. They were able to visualize primary lesions and all main metastatic sites, especially from bladder cancer patients (**Figure 4B**). Furthermore, ^89^Zr-atezolizumab tumor uptake was highly correlated to patient response to atezolizumab as measured by RECIST categorization and Kaplan-Meier curves at a much more significant degree compared to two separate FDA-approved clinical IHC protocols for staining tumor tissues for PD-L1 (**Figure 4C**), strengthening the role that non-invasive imaging can play as a predictive and prognostic marker for anti-PD-L1 immunotherapy. Avelumab, another FDA-approved anti-PD-L1 antibody used for treatment of urothelial and Merkle cell carcinoma, was recently tested in a preclinical setting as a targeting PET tracer 113. Nude immune compromised mice bearing MDA-MB-231 breast cancer cells were injected with ^89^Zr-labeled avelumab and images were obtained 1, 2, 3, and 6 days post-injection. PET images revealed tumor uptake but, unlike ^64^Cu-atezolizumab, high uptake was also observed in many other sites including the blood, lungs, femur, lymph nodes, spleen and liver. *Ex vivo* biodistribution analysis uncovered very high uptake in similar sites. This can be attributed to the fact that Avelumab, unlike Atezolizumab, cross-reacts with murine PD-L1, and thus murine organs that are PD-L1^+^ show specific uptake.

PD-L1 targeting antibodies have proven to be effective whole-body imaging agents translatable into the clinic, however, on-target off-tumor effects, such as PD-L1 staining of epithelial cells or macrophages in distant inflammatory regions, can increase background. To provide higher specificity, a study developed a PD-L1 “pro-antibody” 114. Pro-antibodies, or probody, are antibodies that are only capable of binding to its antigen when a protease cleaves off the peptide mask covering the antigen-binding domain. The protease cleavage site can be engineered to be specific for any protease and, hence, the antibody can be site-specific depending on where in the body the protease is expressed. The first probody was developed targeting VCAM1 to target aortic plaques in a mouse model of atherosclerosis with a matrix metalloprotease 1 (MMP-1) cleavage site, a protease expressed in these plaques 115. They found high staining of aortic plaques when using the probody with no staining in peripheral organs, unlike the parental control antibody that had lower aortic staining with high staining in the pancreas, lung, kidney, and intestine, indicating improved specificity when using the probody. This study laid the foundation for the development of an anti-EGFR probody, the first probody to treat cancer cells 116. They found the probody had similar killing potential compared to the parental control but had a much better preclinical toxicology profile in cynomolgus monkeys. Higher specificity and lower toxicity of probodies are useful features for PET imaging agents. Therefore, a study utilized a newly developed probody, ^89^Zr-CX-072, targeting PD-L1 as a PET imaging agent 114. They found the probody had higher uptake in MDA-MB-231 implanted tumors compared to the parental non-probody antibody. More strikingly, they found much lower tracer uptake, both through imaging and *ex vivo* biodistribution studies, in the spleen as well as the skin, bone, and liver, indicating higher specificity and sensitivity. These results have led to the initiation of a clinical trial to test ^89^Zr-CX-072 in patients with advanced or recurrent solid tumors or lymphomas (2016-002490-36).

Smaller molecules have also been tested in patients to image PD-L1 expression on tumors and predict response to immunotherapy. Adnectins, ~10 kDa in size, are proteins developed based on the framework of the human 10th fibronectin type III domain (^10^FN3). The ^10^FN3 framework is structurally similarity to antibody variable domains and are suitable for PET imaging owing to their small size, favorable pharmacokinetic properties, and a non-toxic non-immunogenic profile 117. An adnectin against PD-L1 has been developed and tested in a preclinical and clinical setting. Preclinical studies showed that anti-PD-L1 radiolabeled adnectins had high signal to background ratio and good bio-distributional properties, with uptake mainly in the tumor, liver, and kidneys 118,119. A ^18^F-labeled adnectin targeting human PD-L1 (BMS-986192), has recently been tested in non-small lung cancer patients in a first-in-human clinical trial, confirming a correlation between tumor uptake of the tracer and both PD-L1 expression using IHC and clinical response to Nivolumab, a PD-1 blockade therapy (**Figure 4D**) 120. Trials in melanoma patients are currently ongoing (NCT03520634).

In conclusion, these studies have illustrated the utility of whole-body imaging of PD-L1. Two first-in-human clinical studies determined significant correlation between PD-L1 detection using PET imaging and response to immunotherapy. Due to the advantage of imaging the entire tumor burden including all metastatic lesions as well as detecting heterogeneous expression of PD-L1 within each tumor, PET imaging is a superior predictive tool versus IHC staining of tumor biopsies. Hence, imaging whole-body PD-L1 expression shows immense promise in not only characterizing the entire tumor burden, but also helping with decision making and patient management, as well as assessing therapeutic response and clinical outcome.

### PD-1

PD-1 is an immune checkpoint molecule expressed on lymphocytes including T cells 121. Anti-PD-1 blocking antibodies, such as pembrolizumab and nivolumab, have shown promising results in the treatment of several advanced malignancies such as metastatic melanoma, non-small cell lung cancer, and Hodgkin's lymphoma, among others 122. Many findings have determined tumor infiltrating T-cells expressing PD-1 are functionally impaired, with PD-1 blockade restoring activity, suggesting noninvasive imaging of PD-1 expression and localization into the tumor can be a clinically relevant predictive marker for anti-PD-1 therapy.

Several preclinical PET studies using murine cancer models were performed to assess the feasibility of imaging PD-1-expressing cells. Animals implanted with B16 melanoma tumors were imaged with a ^64^Cu-DOTA-mouse anti-PD-1 IgG (clone J43) PET tracer that was able to detect the tumor 48 hours post-injection with noticeable tracer uptake in the blood, spleen, kidney, and liver 123. Another study used a ^64^Cu-NOTA-mouse anti-PD-1 IgG2a (RMP1-14) PET tracer and found similar results with high uptake in the tumor, blood, liver, kidney and spleen 24 hours post-injection 100. Given that these PET imaging studies used murine antibodies and cancer cells, subsequent preclinical studies used clinically-relevant human targeting agents and models.

Several studies have used radiolabeled pembrolizumab and nivolumab, FDA-approved anti-human PD-1 IgGs, for PET imaging in humanized mouse models. One study assessed the pharmacokinetic and biodistribution of ^89^Zr-labeled pembrolizumab and found that immune-compromised NSG mice engrafted with human peripheral blood mononuclear monocytes (PBMC) showed higher tracer uptake in the liver and salivary gland 7 days post-injection compared to non-engrafted mice 124. *Ex vivo* immunofluorescence (IF) staining-verified PD1^+^ T-cells were present in the salivary glands, which is a hallmark of graft vs. host disease and other autoimmune diseases 125. A subsequent study extended these experiments to include human A375 melanoma implanted in NOD-SCID immunocompromised mice engrafted with human PBMCs. The study found that uptake of both ^89^Zr- and ^64^Cu-labeled pembrolizumab in the tumor was ~2-fold higher compared with non-engrafted mice 126. Biodistribution analysis revealed that ^89^Zr-pembrolizumab had a lower uptake in peripheral organs compared to ^64^Cu-pembrolizumab while maintaining high uptake in the tumor, kidney, and liver. These preclinical studies led to the initiation of clinical trials using ^89^Zr-pembrolizumab to image patients with NSCLC or metastatic melanoma (NCT02760225, NCT03065764). Additionally, a study utilizing PET imaging with ^89^Zr-nivolumab in NOD-SCID mice implanted with A549 human NSCLC cells 7 days post-injection showed that tracer uptake by the tumor was ~3-fold higher in mice reconstituted with human PBMCs compared to non-grafted mice, a better signal compared to ^89^Zr-pembrolizumab 127. *Ex vivo* biodistribution analysis revealed high uptake of the tracer in the tumor, salivary glands, and spleen, a similar profile to that of ^89^Zr-pembrolizumab. ^89^Zr-nivolumab was also utilized in a clinical study with advanced NSCLC patients in parallel with ^18^F-BMS-986192 (anti-PD-L1 adnectin) 120. PET imaging revealed T-cell infiltration into tumors that were PD-L1+, indicating a correlation between PD-L1 and PD-1 tracer uptake (**Figure 4D**). Furthermore, NSCLC cancer patients responding to nivolumab had ~1.5-fold increase in ^89^Zr-nivolumab uptake in the tumor compared to non-responders, indicating PD-1 imaging can serve as a predictive tool for response to immunotherapy.

While anti-PD-L1 imaging agents mainly label PD-L1-expressing tumor cells, PET imaging using anti-PD-1 targeted agents will label exhausted and activated T cells. Preclinical studies determined the feasibility of imaging T cells infiltrating into tumors, while a clinical study correlated tracer uptake in the tumor to anti-PD-1 therapy response. While these findings are appealing and promising, some concerns remain. Although PD-1 is mainly associated with activated T cells, PD-1 expression has been observed on activated B cells 128 and macrophages 129, indicating that tracer uptake cannot be specifically attributed to T cells. Therefore, to solidify PD-1 expression as a predictive or prognostic marker for immunotherapy, further PET imaging clinical studies are needed.

### CTLA-4

CTLA-4, similar to PD-1, is an inhibitory molecule whose expression is induced on cytotoxic T cell upon activation (94) and is constitutively expressed on Treg cells 130. Similar to PD-1 blockade, CTLA-4 blockade can result in autoimmune-related adverse effects in many patients (130) and, thus, there is an urgent need to develop methods to assess or even predict patient response to CTLA-4 blockade therapies. Clinical studies using patient samples have correlated CTLA-4 expression of tumor-infiltrating T cells to ipilimumab response in melanoma using whole-exome RNA sequencing, and in NSCLC patients through IHC staining of tumor sections 132,133. Thus, imaging of CTLA-4 expressing T cells can be a predictive marker for response to CTLA-4 blockade. Furthermore, monitoring the trafficking of exhausted and/or activated T cells as well as Treg cells in the TME can provide further insight into the behavior of these immune cells. Therefore, PET imaging of CTLA-4 can be both clinically relevant for patients and helpful for expanding our understanding of immunological processes.

An early preclinical PET imaging study used a ^64^Cu-DOTA-anti-murine CTLA-4 IgG2a antibody to visualize T cells in a murine CT26 colon cancer model in BALB/c mice as to evaluate the prospect of imaging CTLA-4 134.* Ex vivo* biodistribution studies revealed a modest yet significant increase (~1.3-fold) in tracer uptake in the tumor compared to IgG control, as well as uptake in the liver and kidney. CTLA-4 expression in tumors was verified to be specific to T-cells and not the tumor cells using RT-PCR, indicating that the PET tracer signal from the tumor is specifically due to tumor-infiltrating T cells. Since the tracer signal in the murine cancer model was not ideal, a subsequent PET imaging study was performed using ipilimumab, a FDA-approved anti-human CTLA-4 IgG1, in humanized mice. This study used ^64^Cu-NOTA-ipilimumab as well as the ^64^Cu-NOTA-ipilimumab-F(ab')2 fragment in NOD-SCID mice engrafted with human PBMCs 135. As expected, both tracers successfully labeled activated T-cells in the salivary glands with ipilimumab-F(ab')2 clearing at a faster rate than the full-sized antibody, while *ex vivo* biodistribution studies showed high uptake in expected sites such as the liver, spleen, and kidney. Thus, this study verified ipilimumab as a valid targeting PET imaging agent that tracked human T cells to the predicted organ site. Although no study has been conducted using human cancer cells implanted in mice engrafted with human PBMCs, the available studies cleared the path for a clinical trial currently being conducted to assess the uptake and biodistribution of ^89^Zr-ipilimumab in metastatic melanoma patients (NCT03313323).

Treatment with CTLA-4 blockage has shown immense promise in melanoma patients yet only a portion of patients respond and over 80% of patients experience some level of adverse effect 136. Thus, there is a need to identify patients who may respond to CTLA-4 blockade. Preclinical studies are promising in that they verified CTLA-4 as an imaging marker of activated T cells. However, preclinical or clinical studies have yet to determine its predictive or prognostic ability. Therefore, further studies need to be conducted to ascertain the clinical relevance of imaging CTLA-4.

### LAG-3

CD223, known as the “lymphocyte activation gene-3” (LAG-3), is another checkpoint molecule present on the surface of T cells 137. Several clinical trials targeting LAG-3 for cancer therapy are ongoing 137. While much progress has been made in targeting LAG-3 for therapy, imaging of LAG-3 is an emerging field. A recent study developed several anti-LAG-3 nanobodies and used ^99m^Tc labeled nanobodies for *in vivo* imaging 138. Animals implanted with murine TC-1 lung epithelial cells, engineered to overexpress LAG-3 were used to establish the imaging. A high tumor-specific signal was observed 1 h post-injection of the radiolabeled nanobody. There is still a need for studies to assess whether LAG-3 imaging can be used to detect tumor-infiltrating lymphocytes and predict the response to LAG-3 or other checkpoint blockades.

Taken together, imaging checkpoint molecules have shown great promise in providing valuable information on the TME, with several imaging probes already moved into the clinical phase. Further studies are needed to better understand how imaging checkpoint molecules can help to make informed decisions on best treatment strategies.

## Imaging engineered T cells

**Figure 5:**
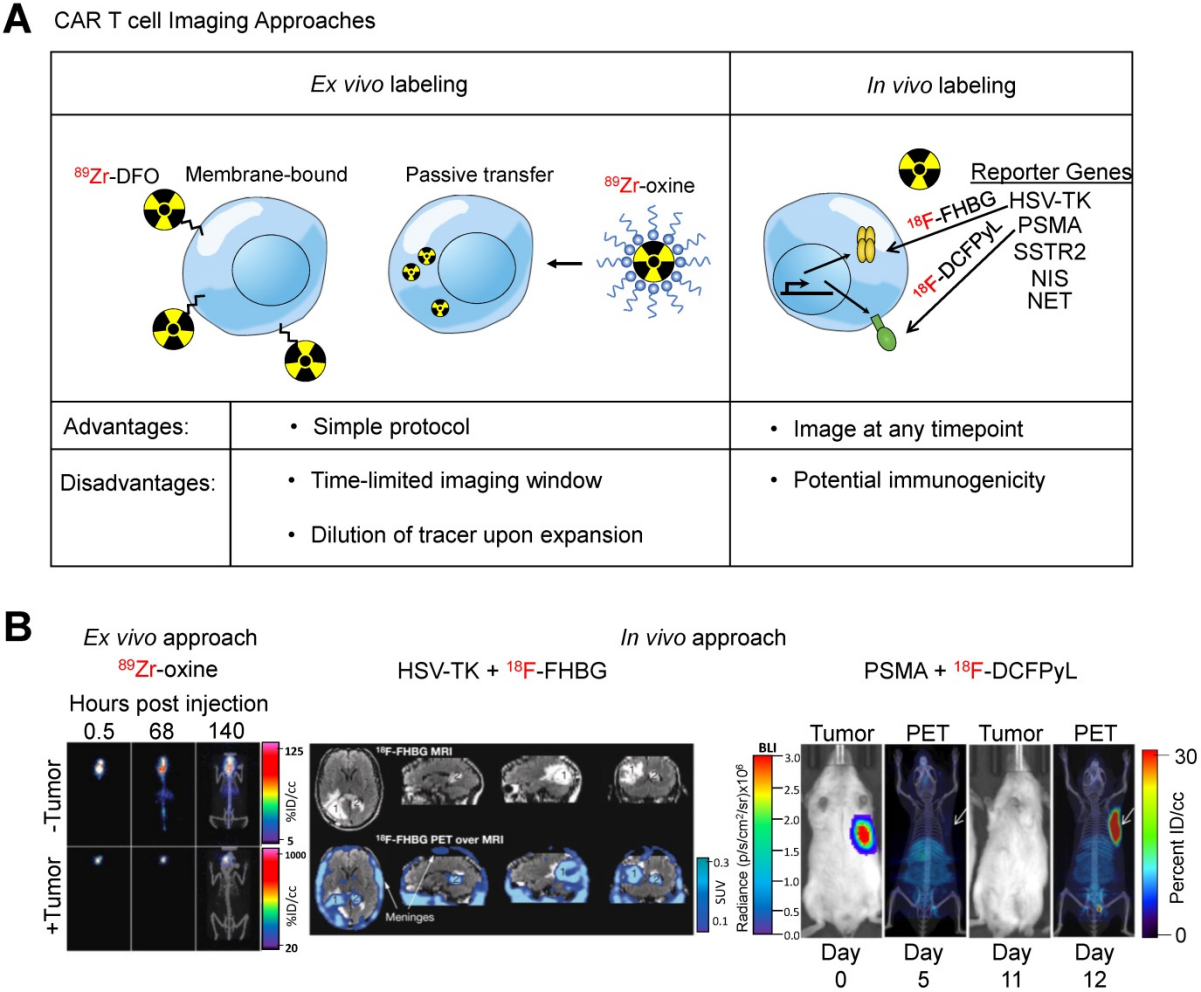
** Imaging Engineered T Cells. (A)** Schematic of different approaches utilized for imaging CAR T cells. Two different approaches are commonly used to label, noninvasively track, and monitor engineered T cells. The *ex vivo* approach involves direct use of the PET radiotracer on the engineered T cell, either through binding of the tracer to proteins on the plasma membrane or through passive transport across the plasma membrane using an ionophoric chelator. The advantage to this method is that it is well-established, simple, and easy to conduct, however it requires same-day imaging due to dilution of the tracer upon T cell expansion. The *in vivo* approach utilizes the expression of a reporter gene in the engineered T cell and, subsequently, infusion of the PET radiotracer specific to the reporter in the patient at a later timepoint. The advantage to this approach is the ability to image at any timepoint. **(B)** Examples of both CAR T cell imaging approaches. An *ex vivo* labeling approach (*left*) illustrates CAR T cells localizing to the GBM tumor even after 140 h after adoptive transfer. Adapted from 148. An *in vivo* labeling approach using the HSV-TK reporter system (*center*) shows the infused CAR T cells localizing a resected tumor 1 and trafficking to a distal tumor site 2. Adapted from 154. A preclinical *in vivo* labeling approach using the PSMA reporter system (*right*) illustrates CAR T cell infiltration into the tumor. Adapted from 168.

Chimeric antigen receptor (CAR) T cells are genetically modified T-cells that express a synthetic receptor capable of binding to a target. The binding to the target cells will result in activation, expansion, and, ultimately, killing of the target cells. CAR T cells have shown promising responses against a variety of blood-born malignancies. For example, CD19-targeting CAR T cells have shown outstanding response in patients with large B cell lymphoma or acute lymphoblastic leukemia (ALL) 139,140. Notwithstanding these encouraging results, responses remain heterogeneous among patients. Many patients show a partial or no response to treatment. In some cases, there is a lack of CAR T cell persistence in the patient 141. A robust prognostic method is needed to assess or even predict the response to CAR T cell treatment. Current methods to assess effectiveness of CAR T cells include detection of cytokines 142, digital PCR analysis 143, or flow cytometry on blood samples 144. However, none of these methods provide as much anatomical scope as whole-body imaging techniques such as PET. Therefore, noninvasive imaging of CAR T cells can prove to be a useful prognostic tool. To accomplish this, studies have utilized two different approaches to image CAR T cells (**Figure 5A**): (i) *ex vivo* radiolabeling of engineered T cells or (ii) *in vivo* radiolabeling of engineered T cells through utilization of reporter genes.

### *Ex vivo* labeling

Direct labeling of immune cells isolated from patients is a commonly used labeling technique in which immune cells are incubated *ex vivo* with an imaging agent before being adoptively transferred into the patient. Early efforts in this regard involved MRI-based detection of immune cells. One such report demonstrated high resolution data of tumor-infiltrating cytotoxic CD8^+^ T cells using high-field MRI and superparamagnetic iron oxide nanoparticles 145. This labeling strategy enables simple, rapid, and specific labeling of any chosen immune cell of interest, including CAR T cells. These MRI-based approaches are also detailed in a prior review 146. In this review we focus on nuclear imaging techniques, which are currently considered to have the highest chance of clinical translation.

*Ex vivo* cell labeling relies on the use of ionophore chelators to carry radioisotopes across the plasma membrane and maintain cell viability. The first instance of *ex vivo* labeling was by direct incubation of ^99m^Tc with human monocytes to monitor chemotaxis 147. However, only 50% cellular viability was observed post-labeling. In order to increase viability while maintaining labeling efficiency, ionophore chelators, such as 8-hydroxyquinoline (oxine) or hexamethylpropyleneamine (HMPAO) are used. Labeling of granulocytes with ^111^In-oxine and ^99m^Tc-HMPAO revealed 98% viability with 73% and 44% labeling efficiency, respectively. These studies established the applicability of ionophore chelators, which are used in *ex vivo* CAR T cell imaging studies. A study utilized ^89^Zr-oxine for labeling anti-IL13Rα2 CAR T cells that targeted glioblastoma multiforme (GBM) cells 148. They found labeling efficiency of CAR T cells was 75% while labeled cells retained more than 60% of the ^89^Zr after 6 days *in vitro*. CAR T cells delivered intraventricularly were detectable by PET at least 6 days post-injection within intracranial patient-derived GBM tumors implanted in NSG mice with no effect on CAR T cell-mediated tumor killing (**Figure 5B**), indicating that *ex vivo* labeling maintains CAR T cell function and PET tracer signal *in vivo* throughout a relevant timeframe.

While 8-hydroxyquinoline is an established ionophore chelator for ^89^Zr, a novel labeling agent, ^89^Zr-labeled-*p*-isothiocyanato-benzyl-desferrioxamine (^89^Zr-DBN), was developed. This PET tracer covalently binds to solvent-exposed lysine residues resulting in higher cellular viability post-labeling and, unlike 8-hydroxyquinoline, no effluxion 149. In a recent study, this agent was used to label anti-CD19 CAR Jurkat cells *ex vivo* with 98% radiolabeling efficiency and detectable signal *in vivo*. However in this model, CAR T cell trafficking into the tumor site was not observed 150. This could be due to the use of Jurkat leukemia cells, instead of engrafted human T cells, as well as subcutaneous injection of Raji cancer cells, a Burkitt's lymphoma line, rather than a relevant human solid tumor cell line. Therefore, further studies with appropriate CAR T cells and mouse models are needed to verify the feasibility of using ^89^Zr-DBN as an *ex vivo* imaging agent.

T-cell receptors (TCR) are an attractive target for *ex vivo* labeling due to their constant recycling and turnover, resulting in internalization and accumulation of the PET tracer. A study was able to take advantage of this phenomenon by labeling the chicken-ovalbumin-specific TCR-transgenic CD4^+^ T cells (cOVA-TCRtg-TH1) using a ^64^Cu-labelled TCR-Ova-specific antibody (KJ1-26) to study the migration pattern of antigen-specific T cells. The radiolabeled antibody was incubated with cells for 30 min and internalization was allowed to proceed for 24 h. The study illustrated that this approach resulted in low radiation-induced cellular damage and low radiotracer efflux 151. The radiolabeled T cells yielded high contrast PET images when injected into animals and their migration was successfully tracked to the pulmonary and perithymic lymph nodes upon OVA-induced airway delayed-type hypersensitivity reaction.

All together, these studies have developed *ex vivo* labeling as a simple technique to label and monitor immune cells. Because CAR T cells are produced *ex vivo* and adoptively transferred back into the patient, *ex vivo* labeling is a straightforward addition to a clinical protocol that does not require any extra invasive or preparatory steps for the patient. Therefore, *ex vivo* labeling of CAR T cells is suitable for incorporation into clinical practice. However, the major limitation of this technique is the timepoint of imaging after infusion due to the decay and effluxion rate of the PET tracer. Thus, the time required for CAR T cells to migrate into the tumor site needs to be appropriate for the decay and effluxion rate of the PET tracer. Furthermore, CAR T cells expand upon activation, which causes dilution of the radiotracer resulting in an overall short half-life of *ex vivo* labeling approaches. Macrophages can also phagocytose labeled cells or cellular debris and migrate, causing nonspecific PET signals. Therefore, further preclinical studies are needed to determine the clinical feasibility of *ex vivo* labeling of CAR T cells.

### *In vivo* labeling

Indirect labeling of CAR T cells using a reporter gene is an imaging strategy that can overcome the shortcomings of *ex vivo* labeling. A reporter gene can mark CAR T cells enabling the cell and its progeny to be distinguished from other cells after they undergo migration, homing and expansion, making noninvasive sequential imaging of cell trafficking possible *in vivo*. Established reporter genes include herpes simplex virus thymidine kinase (HSV-TK), human norepinephrine transporter (hNET), sodium-iodide symporter (hNIS), and others 152.

The HSV-TK has been the most extensively used PET reporter gene system. Patient-derived cytotoxic lymphocytes can be transduced with both the CAR and HSV-TK. Upon infusion of the CAR T cells expressing HSV-TK, radiolabeled penciclovir, an inhibitor of HSV-TK, can be administered intravenously, which will then target the CAR T cells. The strength of this system is the specificity of the reporter/inhibitor and the freedom to inject the targeted imaging agent at any timepoint. This system shows high specificity and low background, especially when using a mutant HSV-TK with higher affinity for penciclovir 153. This reporter system was used in a GBM patient using a CAR T cell targeting IL13Rα2 154, an adoptive cell therapy that has passed phase I clinical trials (157). The study was not only able to image CAR T cells in the resected tumor but was also able to label and monitor CAR T cells that trafficked to an adjacent non-resected GBM tumor (**Figure 5B**). In a follow up study with six additional GBM patients, intraventricular injected CAR T cells expressing HSV-TK were found to be effectively imaged in five patients exhibiting a two-fold increase signal compared to baseline while one patient exhibited high background due to a rich vascular supply with a disrupted blood-brain tumor barrier 156.

A concern that exists with the HSV-TK reporter system is its potential immunogenicity. Clinical studies have shown that CAR T cells expressing HSV-TK are safe and functionally intact 157 but long term studies are needed. To circumvent this potential issue, human reporter genes, such as sodium iodide symporter (hNIS) 158, norepinephrine transporter (hNET) 159, and somatostatin receptor 2 (SSTR2) 160 have been used to label CAR T cells. The advantage of hNIS is that it is non-immunogenic, not internalized, and is only functional in living cells 161, while a well-established radioisotope, Technetium-99m pertechnetate (^99m^TcO_4_^-^) commonly used in clinical nuclear medicine can be used to monitor the effect of therapy. However, hNIS is expressed in many normal epithelial tissues and predominantly expressed in many carcinomas 162. SSTR2 is a potentially more suitable reporter marker due to its limited baseline expression in normal tissues and the availability of clinically approved radiotracers such as ^68^Ga-octreotide analogues (^68^Ga-DOTATOC 163 and ^68^Ga-DOTATATE 164). However, SSTR2 internalizes upon interaction with ligands, reducing imaging sensitivity, and is expressed on immune cells, leading to impaired T cell function upon SSTR2 ligation with ^90^Y-DOTATOC 165.

A recent clinically relevant CAR T cell PET imaging modality was developed utilizing PSMA and 2-(3-(1-carboxy-5-[(6-[^18^F]fluoro-pyridine-3-carbonyl)-amino]-pentyl)-ureido)-pentanedioic acid (^18^F-DCFPyL). PSMA is a cell surface protein whose expression is largely limited to the prostate gland 166, making it an ideal reporter candidate. ^18^F-DCFPyL is a highly sensitive and selective probe targeting PSMA with low off-target staining, providing high contrast clinical images 167, and is currently undergoing phase III clinical trial in patients with suspected recurrence of prostate cancer (NCT03739684). This PET imaging system was able to label T cells co-expressing anti-CD19 CAR and PSMA with high sensitivity *in vitro* and *in vivo* and visualized CAR T cell infiltration into primary and metastatic Nalm6 tumors (**Figure 5B**). Interestingly, this study revealed no correlation between CAR T cell prevalence in peripheral blood, which is the current clinical practice, versus CAR T cell infiltration visualized by PET, indicating CAR T cell presence in the peripheral blood may not accurately reflect therapeutic effectiveness and that PET may be a more clinically relevant prognostic tool 168.

These studies demonstrate the power of reporter systems to label, track, and monitor CAR T cells. Unlike *ex vivo* labeling strategies, *in vivo* labeling approaches allow for PET imaging at any timepoint after CAR T cell infusion and is not subject to signal loss due to CAR T cell persistence and expansion. Reporter genes, such as the PSMA and ^18^F-DCFPyL imaging system, can verify trafficking of CAR T cells to tumor and metastatic sites as well as provide better prognostic power compared to serial assessment in the peripheral blood. Taken together, PET imaging of CAR T cells using reporter genes is highly applicable in the clinic and shows promise as a prognostic tool.

## Conclusion and future directions

While immunotherapy is increasingly being used to treat cancer, there remains a need for methods to adequately characterize the entire tumor burden, assess the response to treatment, and predict which patients are likely to benefit from it. Similarly, there is a need to better assess the presence of many cancer-associated markers (i.e., HER2 or PSMA), which are predictive of response to targeted therapies. The current gold standard procedure for tumor analysis, a tumor biopsy followed by immunohistochemistry, is invasive and has many limitations. Stained samples reflect a small portion of the tumor, and are often unrepresentative of the remainder of the primary tumor or its metastatic lesions 18. Whole body imaging techniques such as PET or SPECT, however, are noninvasive and enable visualization of the distribution markers across the entire tumor burden.

Monitoring the dynamics of immune cells in the TME can help assess or even predict the response to immunotherapy. A number of methods to detect immune cell subsets have been developed, including several that have progressed into clinical trials. These include small molecule probes, as well as antibody and antibody fragments. We can anticipate the clinical translation of tracers for imaging other immune cell markers such as CD11b for myeloid cells and costimulatory molecules such as ICOS for activated T cells in the future.

It is worth noting that nanoparticle-based contrast agents have also been developed to monitor specific immune cell subsets by MRI. Macrophages preferentially phagocytose the iron oxide nanoparticles, allowing for specific contrast enhancement in tumors occupied by TAMs 169. Because of its selectivity for macrophages, ferumoxytol can also be used to assess the macrophage-mediated anti-tumor response. This is particularly interesting in the context of anti-CD47 therapy, where macrophages are activated to phagocytose CD47-expressing cancer cells. In one study, ferumoxytol nanoparticles were successfully used to assess the response to anti-CD47 therapy in a preclinical model of osteosarcoma 170. Nanoparticle-based contrast agents will likely find increasing use as tools to assess the response to immunotherapy and monitor immune cell subsets.

While most studies have focused on imaging of immune cells, imaging of relevant immune cytokines and secreted molecules holds promise as well for assessing the tumor immune status. Imaging of IFN-γ and granzyme B have been successful in assessing the response to immunotherapy in animal models 28,53. There is a need to develop a larger number of cytokine-imaging strategies that can reliably reflect the immune status of the TME in preclinical models and eventually in patients. There are small molecules binding to IL-2 that can potentially be repurposed as PET tracers 171,172. There are still many important targets that have not been imaged in preclinical studies, and we need to better understand how their dynamics correlate with treatment outcome. For example, the presence of chemokines such as CXCL9, CXCL10, and CCL5, have been shown to have prognostic value for the response to immunotherapy 78. Whether imaging such cytokines holds prognostic value remains to be studied. Radiolabeled antibodies or antibody fragments, small molecules, or their cognate receptors, can be useful for imaging such targets.

CAR T cell therapy has been unable to effectively treat solid tumors, which is attributed to the immuno-suppressive environment of many solid tumors. Whole-body imaging of CAR T cells can present a method for assessing response. Each of the two methods used for imaging CAR T cells, *ex vivo* and *in vivo* labeling, provides advantages and disadvantages. Given that T cells need to be extracted from patients, forced to express the CAR, then adoptively transferred back into patients, labeling the cells *ex vivo* is a simple addition to a clinical protocol. However, due to the dilution and efflux of the imaging agent as CAR T cells expand and persist, PET imaging is time restricted. Yet, reporter genes can be expressed in conjunction with the CAR allowing for imaging to be conducted at any timepoint after treatment. Currently, no *ex vivo* labeling is in the clinical phase and there are limited preclinical studies. However, imaging using reporter genes, such as HSV-TK, have been used in the clinic with GBM patients, where CAR T cell tumor infiltration as well as trafficking to distant tumors were observed 27,154. Additionally, preclinical studies have established many other reporter genes, including the promising PSMA and ^18^F-DCFPyL reporter system in CAR T cells 168. These studies indicate that *in vivo* labeling of CAR T cells using reporter genes presents a better method to visualize CAR T cells. A clinical limitation to this method is the potential immunogenicity of the reporter. Although HSV-TK has been shown to be safe 157, long term studies are necessary to assess the immunogenicity of this reporter construct. The human reporter genes SSRT2 and NIS are much less likely to be immunogenic, however they are expressed on other tissues. The most promising preclinical reporter gene is PSMA due to its lack of immunogenicity, lack of basal expression throughout the body excluding the prostate, and easy detection using a high affinity, specific radioisotope. Most strikingly, the study indicated that using PET imaging to assess CAR T cell localization to the tumor lesion is a better indicator of response compared to CAR T cell presence in the blood, indicating a clinical advancement for monitoring CAR T cells using noninvasive whole body imaging. Therefore, these clinical and preclinical PET imaging studies of CAR T cells using reporter systems provide great promise for monitoring clinical response of CAR T cell treatment in the future.

Immune checkpoint blockade therapies, such as PD-1, PD-L1 and CTLA-4 blockade have revolutionized the treatment of cancer. Excitingly, atezolizumab, an FDA-approved PD-L1 blocking antibody, labeled with ^89^Zr has the ability to noninvasively visualize the primary tumor and distant metastatic sites as well as predict the response to atezolizumab treatment at a higher power compared to IHC 112. Although using the same full-sized antibody therapy as a PET imaging agent is highly appropriate, several disadvantages exist. Firstly, a full-sized antibody has a long half-life (3 weeks) and, therefore, a patient is exposed to radiation for a longer period. Secondly, due to its large size, antibodies have low penetrance into the tumor and stays present within circulating blood for a long period of time. This means that patients cannot be imaged the same day upon infusion with the PET tracer. To alleviate these issues, smaller targeted agents such as nanobodies 173, adnectins 120, or peptides 109,174 targeting PD-L1 are being tested in clinical trials to assess their safety and efficacy profiles. These agents have a short half-life, higher penetrance, and faster clearance from circulation, therefore allowing for same day imaging with less radiation exposure to the patient. Hence, these smaller agents are more clinically viable if they can provide the same predictive power as shown with ^89^Zr-atezolizumab.

Unlike imaging PD-L1, PD-1 and CTLA-4 PET imaging are used to assess and monitor T cell activation. This may provide a prognostic tool to determine if PD-1 or CTLA-4 therapy promotes T cell activation and infiltration into the tumor. Further preclinical and clinical studies are necessary to assess their prognostic value. Overall, PET imaging of checkpoint molecules shows great potential in predicting and monitoring patient response to immunotherapy. Further clinical trials can shed light onto the potential scope, such as tumor types, of checkpoint PET imaging. Furthermore, whole-body imaging of immune checkpoints in patients can determine dynamics in checkpoint molecule expression while on immunotherapy and provide insight into the mechanism of resistance or relapse to immunotherapy. Hence, PET imaging of checkpoint molecules provides great utility in both clinical and preclinical applications.

Of note, and in line with the advancement of noninvasive imaging approaches, significant advances are being made in the realm of multifunctional “nanotheranostics” to develop new imaging and therapeutic approaches. Recent nanotheranostic therapeutic advancements include the development of a new liquid brachytherapy approach via novel cationic micelle and liposome formulations 175, and the use of cross-linked iron oxide nanoparticles conjugated to azademethylcolchicine (CLIOT-ICT) as a method to eradicate a subpopulation of quiescent glioblastoma initiating cells 176. Another recent theranostic advancement is the analysis of cancer cells by ultrasensitive dark-field imaging using gold nanoparticle bouquets 177. With a realistic mind regarding a timeline for clinical translation, the above-mentioned approaches may need some additional time to achieve regulatory approval, but represent examples of the next generation of multi-functional agents that are certainly in the pipeline, most of which also make use at least in part of radioimaging or radiotherapy.

Overall, we expect that the efforts outlined in this manuscript will draw the excitement of the field and point to new targets for noninvasive imaging. We anticipate that future mechanistic studies in cancer biology and immunology will reveal novel biological mechanisms, which will point to further potential new targets for imaging. This trend is only expected to grow. Ultimately, the development and clinical translation of such PET tracers will help characterize the entire tumor burden, stratify responders from nonresponders, make informed therapeutic decisions, and assess therapeutic response (or lack thereof) to the benefit of many patients.

## Figures and Tables

**Table 1A:** ImmunoPET Tracers

Target	Agent	Class	Reactivity	Stage	Concluded Clinical Trials	Active clinical trials	References
Macrophage Mannose Receptor (MMR)	^99m^Tc-d a-MMR Nb cl1	Nanobody	Mouse	Preclinical			60
	^18^F-FB-anti-MMR 3.49	Nanobody	Human, Mouse	Preclinical			61
	^68^Ga-NOTA-Anti-MMR-VHH2	Nanobody	Human	Clinical		NCT04168528	62
MHC II	^18^F-FDG-VHH7	Nanobody	Mouse	Preclinical			64
	^64^Cu- VHH4	Nanobody	Human	Preclinical			66
CD3	^89^Zr-DFO-CD3 (clone 17A2)	Intact antibody	Mouse	Preclinical			73,75
CD4	^89^Zr- GK1.5 cDb	Cys-diabody	Mouse	Preclinical			74
ICOS	^89^Zr-DFO-ICOS mAb	Intact antibody	Mouse	Preclinical			83
OX40	^64^Cu-DOTA-AbOX40	Intact antibody	Mouse	Preclinical			84
CD11b	^89^Zr-PEG-DC13	Nanobody	Mouse	Preclinical			78
	^99m^Tc-MAG3-anti-CD11b (clone EP1345Y)	Intact Antibody	Mouse	Preclinical			63
CD8	^89^Zr-Df-IAB22M2C	Minibody	Human	Clinical	NCT03107663	NCT03802123	79
	YTS169-^64^Cu-NOTA	Minibody	Mouse	Preclinical			24
	YTS2.43-^64^Cu-NOTA	Minibody	Mouse (Lyt 2.2 allele)	Preclinical			24
Mouse Dendritic Cells	^99m^Tc-Nb-DC2.1	Nanobody	Mouse	Preclinical			67
	^99m^Tc-Nb-DC1.8	Nanobody	Mouse	Preclinical			

**Table 1B:** Cytokine tracers table

Target	Agent	Class	Reactivity	Stage	Concluded Clinical Trials	Active clinical trials	References
IFN-γ	^89^Zr-DFO-AN-18	Intact antibody	Mouse	Preclinical			28
TNF-α	^64^Cu-DOTA-etanercept	Anti-TNF-α drug (Etanercept)	Human, Mouse	Preclinical			90
Granzyme B	^68^Ga-NOTA-GZP	Peptide	Mouse	Preclinical			53
TGF-β	^89^Zr-fresolimumab	Intact antibody	Human	Clinical	NTC01472731		91
IL-2 receptor	^18^F-FB-IL-2	Labeled cytokine (IL-2)	Human, Mouse	Clinical	NCT02922283 (Terminated)	NCT03304223, NCT04163094, NCT02478099	92
	^68^Ga-Ga-NODAGA-IL2	Labeled cytokine (IL-2)	Human, Mouse	Preclinical			93
	^18^F-AlF-RESCA-IL2	Labeled cytokine (IL-2)	Human, Mouse	Preclinical			

**Table 1C:** Small Molecule Tracers

Agent	Target	Stage	Concluded Clinical Trials	Active clinical trials	References
^18^F-FAC	Deoxycytidine kinase (dCK)	Clinical	NCT01180907, NCT01180868		40
^18^F-CFA	dCK	Clinical		NCT03409419	42
^18^F-F-AraG	Deoxyguanosine kinase (dGK)	Clinical	NCT03007719 (Terminated), NCT02323893	NCT04052412, NCT03142204, NCT04186988, NCT03129061, NCT03684655, NCT03367962	45-47
1-L-^18^F-FETrp	IDO	Preclinical			52
^68^Ga-NOTA-GZP	Granzyme B (Mouse)	Preclinical			53

**Table 1D:** Imaging Engineered T cells

	Agent	Method				Stage	References
*Ex vivo*	^89^Zr-oxine	Passive diffusion				Preclinical	148
	^89^Zr-DBN	Cell surface bound				Preclinical	149,150
	**Reporter gene**	**Species**	**Type**	**Properties**	**Agent**	**Stage**	
*In vivo*	Herpes Simplex Virus - Thymidine Kinase (HSV-TK)	Herpes Simplex Virus 1	Enzyme	Kinase causing intracellular containment of PET tracer	^18^F-FHBG	Clinical	27,154
	Glutamate carboxypeptidase 2 (PSMA)	Human	Cell surface enzyme	Enzyme that produces glutamate	^18^F-DCFPyL	Preclinical	168
	Sodium Iodine Symporter (NIS)	Human	Transporter	Symports sodium and iodine	^99m^TcO_4_^-^	Preclinical	158
	Norepinephrine Transporter (NET)	Human	Cell surface receptor	G-protein coupled receptor	^123^I-MIBG, ^124^I-MIBG	Preclinical	159
	Somatostatin Receptor 2 (SSTR2)	Human	Cell surface receptor	G-protein coupled receptor	^68^Ga-DOTATOC	Preclinical	160
	2D12.5/G54C	Murine	Cell surface receptor	Membrane-bound antibody	^86^Y-AABD	Preclinical	178
	Dihydrofolate reductase enzyme (DHFR)	Escherichia coli	Enzyme	Enzyme that produces tetrahydrofolate	^18^F-TMP	Preclinical	179

**Table 1E:** Imaging Checkpoint molecules

Target	Agent	Class	Reactivity	Stage	Concluded Clinical Trials	Active clinical trials	References
PD-L1	^18^F-B3, ^64^Cu-B3	Nanobody	Murine	Preclinical			105
	^99m^Tc-C3, ^99m^Tc-C7, ^99m^Tc-E2, ^99m^Tc-E4, ^99m^Tc-K2	Nanobody	Murine	Preclinical			106,107
	^64^Cu-NOTA-10F.9G2	Antibody IgG2b	Murine	Preclinical			100
	^89^Zr-DFO-10F.9G2	Antibody IgG2b	Murine	Preclinical			103
	^111^In-DTPA-anti-PDL1	Antibody	Murine	Preclinical			180,181
	^68^Ga-WL12	Peptide	Human	Preclinical			182
	^18^F-FPy-WL12	Peptide	Human	Preclinical			183
	^18^F-NOTA-Z_PD-L1_1_	Affibody	Human	Preclinical			108
	^64^Cu-DOTA-FN3_hPD-L1_	Adnectin	Human	Preclinical			118
	^68^Ga-NOTA-Nb109	Nanobody	Human	Preclinical			184
	^64^Cu-DOTA-HAC-PD1	High Affinity PD-1 ectodomain	Human	Preclinical			185,186
	^64^Cu-NOTA-avelumab Fab	Fab	Human	Preclinical			187
	^111^In-labeled atezolizumab	Antibody IgG1	Human	Preclinical			188
	^64^Cu-DOTA-atezolizumab	Antibody IgG1	Human	Preclinical			111,188
	^111^In-PD-L1.3.1	Antibody IgG1	Human	Preclinical			189
	^89^Zr-DFO-C4	Antibody IgG1	Human	Preclinical			190
	^64^Cu-WL12	Peptide	Human	Clinical		NCT04304066	109,174
	^99m^Tc-NM-01	Nanobody	Human	Clinical	NCT02978196		173
	^89^Zr-envafolimab	Nanobody Fc fusion	Human	Clinical		NCT03638804	191,192
	^18^F-BMS-986192	Adnectin	Human	Clinical	2015-004760-11	NCT03520634, NCT03843515, NCT03564197, NCT03843515, 2018-002643-28,	120,193
	^89^Zr-durvalumab	Antibody IgG1	Human	Clinical		2015-005765-23, NCT03829007, NCT03853187	
	^89^Zr-labeled avelumab	Antibody IgG1	Human	Clinical		NCT03514719	113
	^89^Zr-labeled atezolizumab	Antibody IgG1	Human	Clinical	NCT02453984, NCT02478099	NCT03850028, NCT04006522, NCT04222426, NCT02478099, 2019-001197-28, 2017-003511-20	112
	^89^Zr-CX-072	Pro-antibody	Human	Clinical		2016-002490-36	114
PD-1	PD-1-liposome-DOX-^64^Cu	Liposome	Murine	Preclinical			194
	^64^Cu-DOTA-J43	Antibody IgG	Murine	Preclinical			123
	^64^Cu-NOTA-RMP1-14	Antibody IgG2a	Murine	Preclinical			100
	^64^Cu-pembrolizumab	Antibody IgG4	Human	Preclinical			126,195
	^89^Zr-pembrolizumab	Antibody IgG4	Human	Clinical		NCT02760225, NCT03065764, NCT03446911*, 2015-004260-10, 2016-003819-36	124,126
	^89^Zr-nivolumab	Antibody IgG4	Human	Clinical	2015-004760-11		120,127
CTLA-4	^18^F-H11, ^89^Zr-H11	Nanobody	Murine	Preclinical			196
	^64^Cu-DOTA-anti-CTLA-4	Antibody IgG1	Murine	Preclinical			134
	^64^Cu-NOTA-ipilimumab-F(ab')2	F(ab')_2_	Human	Preclinical			135
	^64^Cu-NOTA-ipilimumab	Antibody IgG1	Human	Preclinical			135
	^64^Cu-DOTA-ipilimumab	Antibody IgG1	Human	Preclinical			197
	^89^Zr-ipilimumab	Antibody IgG1	Human	Clinical		NCT03313323, 2012-003616-31	

*PET imaging using radiotracer is considered a secondary outcome.

## Abbreviations

1-L-^18^F-FETrp: 1-(2-^18^F-fluoroethyl)-L-tryptophan
^10^FN3: 10th fibronectin type III domain
^18^F-CFA: 2-chloro-2'-deoxy-2'-[18F]fluoro-9-b-D-arabinofuranosyl-adenine
^18^F-DCFPyL: 2-(3-(1-carboxy-5-[(6-[^18^F]fluoro-pyridine-3-carbonyl)-amino]-pentyl)-ureido)-pentanedioic acid
^18^F-FAC: 1-(2'-deoxy-2'-[^18^F]fluoroarabinofuranosyl) cytosine
^18^F-FB: N-(4-[^18^F]fluorobenzoyl)
^18^F-FDG: 2-deoxy-2-[fluorine-18]fluoro-D-glucose
^89^Zr-DBN: ^89^Zr-labeled-*p*-isothiocyanato-benzyl-desferrioxamine
ACT: adoptive cell therapy
ALL: acute lymphoblastic leukemia
AraG: 9-(β-D-Arabinofuranosyl)guanine
BCMA: B cell maturation antigen
CAR: chimeric antigen receptor
cOVA-TCRtg-TH1: chicken-ovalbumin-TCR-transgenic TH1 cells
CT: computed tomography
CTLA-4: cytotoxic lymphocyte antigen-4
dCK: deoxycytidine kinase
FcRn: neonatal Fc receptor
FLT: fluorothymidine
GBM: glioblastoma multiforme
GvHD: graft versus host disease
HMPAO: hexamethylpropyleneamine
hNET: norepinephrine transporter
hNIS: sodium-iodide symporter
HNSCC: head and neck squamous cell carcinoma
HSV-TK: herpes simplex virus - thymidine kinase
IDO: indoleamine 2,3-dioxygenase
IF: immunofluorescence
IFN-γ: interferon gamma
IHC: immunohistochemistry
IL-2: Interleukin-2
irAEs: immune-related adverse events
MMP-1: matrix metalloprotease-1
MMR: macrophage mannose receptor
MRI: magnetic resonance imaging
NK: Natural Killer
NOD/SCID: non-obese diabetic/severe combined immunodeficiency
Oxine: 8-hydroxyquinoline
PBMC: peripheral blood mononuclear monocytes
PD-1: programmed cell death protein-1
PEG: polyethylene glycol
PET: positron emission tomography
PSMA: prostate specific membrane antigen
RT: radiation therapy
scFv: single chain variable fragment
SPECT: single-photon emission computed tomography
SSTR2: somatostatin receptor 2
STING: stimulator of interferon genes
TCR: T cell receptor
TILs: tumor-infiltrating-lymphocytes
TK1: thymidine kinase 1
TME: tumor microenvironment
TNBC: triple negative breast cancer
TNF-α: tumor necrosis factor alpha
TPA: 12-O-tetradecanoyl-phorbol-13-acetate
